# Predictors of Dizziness and Hearing Disorders in People with Long COVID

**DOI:** 10.3390/medicina59111901

**Published:** 2023-10-26

**Authors:** Faten S. Obeidat, Alia A. Alghwiri, Susan L. Whitney

**Affiliations:** 1 Department of Hearing and Speech Sciences, School of Rehabilitation Sciences, University of Jordan, Amman 11942, Jordan; 2 Department of Physiotherapy, School of Rehabilitation Sciences, University of Jordan, Amman 11942, Jordan; 3 Department of Physical Therapy, School of Health and Rehabilitation Sciences, University of Pittsburgh, Pittsburgh, PA 15260, USA; 4 Department of Otolaryngology, School of Medicine, University of Pittsburgh, Pittsburgh, PA 15260, USA

**Keywords:** dizziness, hearing loss, COVID-19, long COVID, predictors, fatigue, quality of life, fear of falling, neurology

## Abstract

*Background and Objectives*: Individuals report persistent symptoms after becoming infected by SARS-CoV-2 (COVID-19) that last for >4 weeks (long-COVID syndrome). Dizziness and hearing loss have been reported among long-COVID symptoms. However, little is known about the potential predictors of dizziness and hearing loss in individuals with long COVID. This study aimed to explore the presence and correlates of dizziness and hearing loss in a sample of people with long-COVID syndrome. *Materials and Methods*: Individuals aged 18 years and older who were infected with COVID-19 at least 8 weeks prior to the start of the study were included if they were not diagnosed with dizziness or hearing loss before getting COVID-19. Demographics and COVID-19-related information were collected. Participants completed the Dizziness Handicap Inventory (DHI), Activities-Specific Balance Confidence (ABC) scale, Falls Efficacy Scale International (FES-I), Modified Fatigue Impact Scale (MFIS), and Medical Outcomes Study Short Form 12 (SF-12). Finally, hearing was assessed using pure-tone audiometry (PTA) in a subsample. *Results*: Two hundred and nine individuals (66% female) with a mean (SD) age of 27 (9) participated in the study. Perceived dizziness and hearing loss were reported in 26 and 15.3% of the sample, respectively. Logistic regression was conducted to identify potential predictors of dizziness and hearing loss separately. After controlling for age and severity of dizziness, female sex and high fatigue severity were associated with an increased likelihood of reporting dizziness (R^2^ = 31%). The severity of dizziness and neurological symptoms during the acute stage of COVID-19 were associated with an increased likelihood of reporting hearing loss (R^2^ = 10.4%) after controlling for age. *Conclusions*: Dizziness and hearing loss present in long COVID and can be disabling. Females with high levels of fatigue should be questioned about persistent dizziness. Hearing loss should be considered in individuals with neurological symptoms and severe dizziness as a consequence of long COVID.

## 1. Introduction

During the active phase of coronavirus disease 2019 (COVID-19), people may present with various symptoms related to the respiratory system such as fever and cough as well as other symptoms such as fatigue and musculoskeletal pain [1]. However, some symptoms persist or new symptoms emerge after the end of the acute and subacute phases of the first 4 weeks without a clear explanation, leaving patients in an abnormal state of health [2]. Experiencing residual symptoms beyond 4 weeks of having COVID-19 is considered a syndrome and has been called long COVID [3]. Long COVID has been found to affect the individual’s quality of life (QOL) and interfere with their activities and participation 35 days after their infection [4].

The understanding of the nature of long COVID is still growing. Interestingly, it was reported that long-COVID symptoms are highly variable in their number and presence during the course of the day since patients may experience a single symptom or multiple symptoms that are either constant or fluctuate throughout the day [3]. Several investigations have been conducted to understand the hypotheses behind long-COVID syndrome [5,6,7]. Long COVID may be explained based on four hypotheses including variants of the virus, responses from the individual’s immune system, inflammation, or oxidative stress [5].

Long COVID or the post-COVID-19 condition can affect any individual infected by the COVID-19 virus, irrespective of the severity of the acute symptoms [8]. Moreover, long-COVID symptoms may include disorders to different organs and systems. Long-COVID symptoms have been classified into seven systems including immune, hematological, pulmonary, cardiovascular, gastrointestinal, skeletal, and the nervous system [9]. Common symptoms of long COVID include fatigue, respiratory disorders, cognitive disorders, depression, anxiety, dizziness, and hearing loss [10,11].

Dizziness, a nonspecific term that may denote light headedness, vertigo or imbalance, was reported as one of the neurological symptoms during acute COVID-19 by several studies [12,13,14]. One of the earliest studies in China found that dizziness was the most common neurological manifestation after COVID-19 [15]. Further studies investigated the prevalence and severity of dizziness in long COVID [16,17]. Based on a recent systematic review and meta-analysis, the prevalence of dizziness in long COVID was reported in approximately 5% of 2219 individuals [17].

Hearing loss due to COVID-19 infection has received little attention in the literature [18,19,20]. Hearing centers in the temporal lobe of the brain may be affected by the COVID-19-virus infection causing permanent hearing damage [18]. A retrospective study conducted by Swain et al. (2021) reported that 2.5% of study participants with COVID-19 were diagnosed with sudden sensorineural hearing loss (SNHL) confirmed by pure tone audiometry (PTA) [20]. A systematic review included 30 studies which investigated hearing loss in patients with positive COVID-19 of which 29 studies reported sudden SNHL with an estimated prevalence of 7.6% [21].

Dizziness and hearing impairments in long COVID were reported as common neurological symptoms that may indicate an invasion of the virus to the inner ear or vestibulo-cochlear cranial nerve [9]. However, no studies have investigated both dizziness and hearing impairments in long COVID. Therefore, the aim of this study was to detect the perceived dizziness and hearing impairments in people with long COVID and their potential predictors. 

## 2. Materials and Methods

A cross-sectional-study design was implemented between September 2022 and April 2023 to investigate the presence and correlates of dizziness and hearing loss in people with long COVID. Flyers about the research project were posted at the University of Jordan to recruit subjects. The majority of our sample were university students. Ethics approval was sought from the University of Jordan Institutional Review Board (IRB: 5-2022; approved date: 17 January 2022). A consent form was signed by all participants prior to the start of study procedures. Participants were provided with unique codes to ensure their confidentiality. 

### 2.1. Participants

People aged between 18 and 60 years old who had had COVID-19 at least 8 weeks before the beginning of the study were included. We included individuals who were infected by COVID-19 8 weeks before the study because most of the outcome measures used in this study ask about their symptoms in the previous month. People who were older than 60 years old were excluded since hearing disorders are common after the age of 60 [22]. Furthermore, individuals who had dizziness, imbalance, or hearing loss before getting COVID-19 were also excluded using the following question (have you had dizziness, imbalance, or hearing loss before getting infected by COVID-19?).

### 2.2. Procedures

Recruitment took place at the University of Jordan where the primary investigator met potential participants where subject eligibility was determined. Eligible individuals based on their inclusion/exclusion criteria were recruited in this study starting with signing the informed consent. The following study information was collected from all participants: demographics and COVID-19-related questions. 

In order to quantify the impact of dizziness on disability level and fear of falling, the Dizziness Handicap Inventory (DHI) [23] and Falls Efficacy Scale International (FES-I) [24] were examined, respectively. Additionally, to quantify the severity of fatigue and the health-related quality of life, the Modified Fatigue Impact Scale (MFIS) [25] and Medical Outcomes Study Short Form 12 (SF-12) [26] were completed, respectively. Finally, a random sample of participants underwent tympanometry and pure-tone audiometry (PTA) to evaluate middle ear function and hearing, respectively, which were conducted by a specialized audiologist in a sound-treated room.

### 2.3. Outcome Measures

Age, sex, marital status, body mass index (BMI), time since diagnosed with COVID-19 (duration in months), symptoms during acute COVID-19 (respiratory, neurological, and gastrointestinal), and finally a question about perceived dizziness or hearing loss as a long-COVID symptom (yes or no).

The Dizziness Handicap Inventory (DHI) is a self-report questionnaire that has been used to quantify the level of disability due to dizziness symptoms [23]. The total score of DHI ranges between 0 “no dizziness” and 100 “severe dizziness”. The DHI has good psychometric properties [23] and was translated into and validated in the Arabic language [27].

The Falls Efficacy Scale International (FES-I) is a self-report questionnaire that quantifies the individual’s perception of fear of falling during various activities of daily living [24]. The total score of FES-I ranges between 16 and 64 with a higher score encompassing a worse perception of fear of falling. The FES-I has good psychometric properties and was translated into and validated in the Arabic language [28,29].

The Modified Fatigue Impact Scale (MFIS) is a self-report questionnaire that quantifies the physical, cognitive, and psychological impact of fatigue symptoms [25]. The total score of the MFIS ranges between 0 and 84 with a higher score indicating greater severity of fatigue symptoms. The MFIS has good psychometric properties [30]. The content of MFIS was explored using the International Classification of Functioning, Disability and Health (ICF), and was found to focus on body structures and activities and participation components of the ICF [31]. Moreover, the MFIS has been translated into and validated in the Arabic language [32].

The SF-12 is a self-report questionnaire that quantifies physical, mental, and global quality of life (QOL) [26]. The global score ranges between 0 and 100 with a higher score indicating a better QOL. The SF-12 has good psychometric properties [33] and has been translated into and validated in Arabic [34].

Tympanometry was performed before PTA to rule out middle ear pathologies. Patients with type-B, type-As, type-AD and type-C tympanograms were excluded from the study. Tympanometry was carried out using an Interacoustic AT235^®^ device (5500 Middelfart-Denmark). 

PTA measurements were performed using an Interacoustics AC40 Clinical Audiometer. Air conduction (AC) thresholds were measured in the range of 250–8000 Hz using Telephonics TDH39 earphones and bone thresholds were measured in the range of 500–2000 Hz using B71 bone vibrator. PTA test was performed by a specialized audiologist based on the British Society of Audiology recommended procedures [35] for both ears in participants. The average of PTA results for each ear was reported in the data for statistical analysis. The average of the pure-tone AC hearing thresholds at 250, 500, 1000, 2000, and 40,000 Hz was calculated [35]. An average of PTA greater than 20 decibels was considered as hearing loss in the current study [35].

### 2.4. Statistical Analysis

Descriptive and inferential statistics were conducted to analyze study variables. The responses to the question about perceived dizziness/hearing loss as a long-COVID symptom was the dependent variable. Mann–Whitney U test was used to assess the differences in study variables between people with and without perceived dizziness/hearing loss. A binary logistic regression was conducted to identify potential predictors of perceived dizziness/hearing loss. SPSS v.28 was used for the analysis and a *p* < 0.05 was considered significant.

## 3. Results

A sample of 209 individuals with a mean (SD) age of 27 (9) and 66% females participated in the study. Most of study sample were university students and university staff. Perceived dizziness was reported in 26% and perceived hearing loss was reported in 15% of the participants. The remaining sample characteristics are presented in Table 1. 

### 3.1. Perceived Dizziness

The differences in study variables between participants with and without perceived dizziness are shown in Table 2. Using the logistic regression, female sex and higher fatigue levels were associated with increased likelihood of reporting dizziness (R^2^ = 31%) after controlling for age and the severity of dizziness (DHI) (Table 3).

### 3.2. Hearing Loss

The differences in study variables between participants with and without perceived hearing loss are shown in Table 4. Using logistic regression, the severity of dizziness and having neurological symptoms in acute COVID-19 were associated with an increased likelihood of reporting hearing loss (R^2^ = 10.4%) after controlling for age (Table 5).

A subsample of 160 participants underwent PTA testing. The PTA mean (SD) for the right and left ears was 6.66 (8.57) and 6.49 (9.01), respectively. The abnormal PTA mean (SD) for right and left ears was 35.6 (10.1) and 39.7 (12), respectively. The normal PTA mean (SD) for right and left ears was 4.7 (4.3) and 4.96 (5.1), respectively.

## 4. Discussion

The aims of this study were to explore the persistence of dizziness and hearing loss symptoms in a sample of people after COVID-19 and to determine the potential predictors of dizziness and hearing loss as long-COVID symptoms. To our knowledge, this is the first study that identifies potential predictors of dizziness and potential predictors of hearing loss in people with long COVID.

### 4.1. Dizziness in Long COVID

In our study, of 209 individuals who had COVID-19, 26% reported dizziness as a persistent symptom after COVID-19. Although other studies found different prevalences of dizziness during the acute stage of COVID-19 [15] or long COVID (5%) [17], a recent systematic review and meta-analysis reported a pooled dizziness prevalence of 26.4% [36] which is similar to our finding.

When study variables were compared between the participants with and without perceived dizziness after COVID-19, several factors had significant differences. Women had worse perceived dizziness than men with long COVID. This finding is consistent with previous studies that reported a strong association between dizziness and female sex [37,38,39]. The health records of a nationally representative sample from the United States were reviewed and found that 66% out of 221,273 patients who suffered from dizziness or vertigo were females [38]. A prospective study that was conducted in Germany and included more than 7 million individuals reported that females (65.4%) were significantly more affected by dizziness than males, especially between 15 and 75 years of age [39]. The pathophysiology behind this finding is not fully understood; however, potential explanations have been proposed. One of the potential reasons might be the higher number of comorbidities from which females suffer, such as anxiety disorders, that contribute to dizziness [40,41]. Another suggested explanation was the hormonal changes in younger adults and after menopause [42]. The higher incidences of vitamin D deficiency and osteoporosis in older women were suggested as risk factors for the diagnosis of benign paroxysmal positional vertigo [43].

The percentage of people who had respiratory or neurological symptoms during the acute stage of COVID-19 was significantly higher in individuals with perceived dizziness in long COVID than people without dizziness. This finding can be explained by the use of COVID-19 medications during the acute stage due to the presence of respiratory or neurological impairments which may have vestibular-toxic effect [44]. The utilization of high doses of COVID-19 medications in a short period may have a toxic effect on the vestibular system that could manifest as dizziness and imbalance afterward.

Perceived disability due to dizziness, fear of falling, and fatigue severity at the time of the study were all significantly worse in individuals with perceived dizziness in long COVID compared to people without dizziness. Fatigue was found as a common symptom in long COVID along with dizziness [45]. The new finding in this study was that fatigue was associated with people who reported dizziness more than with people without dizziness. Therefore, the presence of dizziness and fatigue appears to coexist in long COVID.

Fear of falling has been found to be associated with dizziness in older adults [46]. Another study found that the severity of dizziness was one of the predictors of fear of falling even in younger adults who suffer from peripheral vestibular hypofunction [47]. Consequently, the current finding of a substantial relationship between fear of falling and perceived dizziness as persistent symptoms of long COVID in younger adults highlights the similarity between dizziness as a symptom of vestibular disorders and fear of falling. 

Moreover, QOL was significantly better in individuals without dizziness than in people with perceived dizziness in long COVID. This finding is consistent with other studies that found a significant association between QOL and dizziness in different populations such as older adults [48] and people with vestibular disorders [49]. In a systematic review, individuals who reported dizziness in long COVID indicated that the severity of their dizziness varied from mild to severe which affected their QOL [21]. A recent study highlighted the disabling effect of dizziness and headache in long COVID and found that these were significant predictors of dependence in individuals with long COVID [50]. Individuals who suffered from dizziness and headache as long-COVID symptoms reported lower functional ability and worse disability levels in performing their activities of daily living such as self-care, home management, employment and recreation, shopping, communication, and travel [50].

In this study, female sex and higher fatigue levels were found to be potential predictors of reporting dizziness as a long-COVID symptom. This indicates that females with higher levels of fatigue should be questioned about persistent dizziness after COVID-19 and may require greater attention from health care providers for early detection and proper management of dizziness.

### 4.2. Hearing Loss in Long COVID

Of the 209 individuals who had COVID-19, 15.3% reported hearing loss as a persistent symptom from COVID-19. The perception of hearing loss was high (15.3%) compared to other studies that found it around 7.6% [21]. However, SNHL findings using PTA in a subsample (n = 160) of this study was 9.4% which was lower than the participants’ perceived hearing loss. In a recent study by Dusan et al., tonal audiometry showed SNHL in a high percentage (40.5%) of individuals in the acute stage of COVID-19 [51].

Women had greater perceived hearing loss than men with long COVID. This is consistent with a study by Dusan et al. who found that women had greater hearing loss than men in their COVID-19 group [51]. 

The percentage of people who had neurological symptoms during the acute COVID-19 period was significantly higher in individuals with perceived hearing loss in the long-COVID group compared to people without hearing loss. The predisposition of neurological impairments during the acute stage of COVID-19 indicates that the perceived hearing loss in long COVID may be of a neurological origin. Neurological symptoms during COVID-19 were speculated to be caused by an invasion of the virus to the peripheral or central nervous system, which in our case may include the inner ear [9] or hearing centers in the brain [18].

Perceived disability due to dizziness, fear of falling, and fatigue severity at the time of the study were all significantly worse in individuals with perceived hearing loss in long COVID than people without hearing loss. None of the previous studies evaluated the prevalence of perceived disability due to dizziness, fear of falling, and fatigue severity in patients with perceived hearing loss in long-COVID survivors. 

The severity of dizziness and having neurological symptoms in acute COVID-19 were predictors of reporting hearing loss as a long-COVID symptom. Therefore, it is recommended to conduct routine hearing screening for all COVID-19 positive cases for an early diagnosis and immediate treatment for COVID-19 patients [20]. There is a need for more studies to perform a comprehensive assessment of the hearing functions in COVID-19 patients including testing for central auditory-processing disorders. 

### 4.3. Dizziness and Hearing Loss in Long COVID

Reporting both perceived dizziness and hearing loss together in long COVID was found in 8.6% of the 209 participants. A higher percentage of the participants who had both perceived dizziness and hearing loss was found to be female. 

Several studies have reported the association between hearing loss and dizziness/vertigo in people with vestibular dysfunction such as Ménière’s disease [52]. However, this is the first study to investigate the relationship between hearing loss and dizziness as long-COVID symptoms. The evidence in the literature concerning the higher incidence of vestibular disorders in females [36,37], along with the finding in this study that a higher number of females reported having both hearing loss and dizziness, may suggest the involvement of the vestibular system in long COVID. However, this warrants further investigation.

### 4.4. Limitations

We have a number of limitations to report in our study. The sample of this study included a high number of young subjects and more females, and mainly university students. This fact may affect the generalizability of the results to middle and older adults and to both sexes. Another limitation is the nature of the cross-sectional study that does not allow for exploring cause and effect. A longitudinal study starting from the acute stage of COVID-19 up to the long-COVID stage would have provided us with more detailed information about the incidence of dizziness and hearing loss and the possible causes. 

## 5. Conclusions

COVID-19 leads to persistent symptoms that are referred to as long COVID. Several symptoms have been reported in long COVID including dizziness and hearing loss. In this study, we investigated factors associated with dizziness and with hearing loss in individuals with long COVID. Being a female with high levels of fatigue were predictors of reporting dizziness in long COVID. Additionally, having neurological symptoms in acute COVID-19 and dizziness severity were predictors of reporting hearing loss in long COVID. Until now, there has been no definite explanation that clarifies how dizziness and hearing-loss symptoms persist in long COVID. 

## Figures and Tables

**Table 1:** Descriptive statistics of sample characteristics (N = 209).

Characteristics	Males (N = 72)	Females (N = 137)	Total (N = 209)
Age in years, mean (SD)	31.28 (13.21)	24.47 (8.35)	26.81 (10.76)
BMI, mean (SD)	26.19 (5.30)	24.16 (5.07)	24.85 (5.23)
Sex, n (%)			
Male			72 (44%)
Female			137 (66%)
Marital status, n (%)			
Single	41 (56.9%)	105 (76.6%)	146 (70%)
Married	30 (41.7%)	28 (20.4%)	58 (27.7%)
Divorced	1 (1.4%)	2 (1.5%)	3 (1.4%)
Widowed	0	2 (1.5%)	2 (0.9%)
Time since diagnosed with COVID-19 in months, mean (SD)	10.89 (5.14)	10.40 (3.13)	10.57 (3.94)
Symptoms during acute COVID-19, n (%)			
Respiratory symptoms	46 (63.9%)	101 (73.7%)	147 (70%)
Neurological symptoms	27 (37.5%)	76 (55.5%)	103 (49%)
Gastrointestinal symptoms	18 (25%)	39 (28.5)	57 (27%)
Perceived dizziness in long COVID, n (%)			
Yes	9 (12.5%)	46 (33.6%)	55 (26%)
No	63 (87.5%)	91 (66.4%)	154 (74%)
DHI total score, mean (SD) (n = 55) *	37.8 (30)	25.7 (24.1)	27.71 (25.26)
Perceived hearing loss in long COVID, n (%)			
Yes	5 (7%)	27 (80.3%)	32 (15.3%)
No	67 (93%)	110 (67.9%)	177 (84.7%)
Right ear PTA (n = 160), n (%)			
Normal	36 (83.7%)	109 (93.2%)	145 (90.6)
Abnormal	7 (16.3%)	8 (6.8%)	15 (9.4%)
Left ear PTA (n = 160), n (%)			
Normal	39 (90.7%)	109 (93.2%)	148 (92.5)
Abnormal	4 (9.3%)	8 (6.8%)	12 (7.5%)
Reporting both perceived dizziness and hearing loss			
in long COVID, n (%)			
Yes	2 (2.8%)	16 (11.7%)	18 (8.6%)
No	70 (97.2%)	121 (88.3%)	191 (91.4)
FES-I total score, mean (SD)	20.47 (10.24)	22.14 (8.34)	21.56 (9.05)
MFIS total score, mean (SD)	22.47 (19.76)	31.80 (22.84)	28.59 (22.3)
SF-12 total score, mean (SD)	72.42 (16.40)	63.63 (18.20)	66.66 (18.6)

BMI: body mass index; DHI: Dizziness Handicap Inventory; PTA: pure-tone audiometry; FES-I: Falls Efficacy Scale International; MFIS: Modified Fatigue Impact Scale; SF-12: Medical Outcomes Study Short Form 12. * For individuals who reported dizziness.

**Table 2:** Differences in study variables between individuals with and without perceived dizziness using Mann–Whitney U test (N = 209).

Variables	Without Dizziness (N = 154)	With Dizziness (N = 55)	*p*-Value
Age (in years), mean (SD)	27 (10.85)	26.27 (10.58)	0.94
Sex, n (%)			0.001
Male	63 (41%)	9 (16.4%)	
Female	91 (59%)	46 (83.6%)	
BMI, mean (SD)	24.81 (5.13)	24.97 (5.54)	0.71
Time since diagnosed with COVID-19 in months, mean (SD)	10.55 (4.15)	10.62 (3.30)	0.60
Symptoms during acute COVID-19, n (%)			
Respiratory symptoms	100 (65%)	47 (85.5%)	0.004
Neurological symptoms	68 (44%)	35 (63.6%)	0.013
Gastrointestinal symptoms	37 (24%)	20 (36.4%)	0.079
DHI total score, mean (SD)	4.95 (10)	27.71 (25.26)	0.001
FES-I total score, mean (SD)	20 (8.14)	25.89 (9.67)	0.001
MFIS total score, mean (SD)	23.44 (19.73)	43 (22.65)	0.001
SF-12 total score, mean (SD)	71.26 (15.66)	53.77 (18.20)	0.001

BMI: body mass index; DHI: Dizziness Handicap Inventory; PTA: pure-tone audiometry; FES-I: Falls Efficacy Scale International; MFIS: Modified Fatigue Impact Scale; SF-12: Medical Outcomes Study Short Form 12.

**Table 3:** Regression analysis of having dizziness in long COVID.

	Estimates	Standard Error	t Value	*p* Value
Constant	−1.386	0.848	2.672	0.102
Age	0.001	0.023	0.002	0.969
Female sex	−1.127	0.547	4.239	0.040
DHI total score	0.071	0.014	24.307	0.001
MFIS total score	0.026	0.010	7.431	0.006

DHI: Dizziness Handicap Inventory; MFIS: Modified Fatigue Impact Scale.

**Table 4:** Differences in study variables between individuals with and without perceived hearing loss using Mann–Whitney U test.

Variables	Without Hearing Loss (N = 177)	With Hearing Loss (N = 32)	*p*-Value
Age (in years), mean (SD)	23.53 (8.9)	24.9 (10.1)	0.368
Sex, n (%)			0.015
Male	67 (93%)	5 (7%)	
Female	110 (67.9%)	27 (80.3%)	
BMI, mean (SD)	24.5 (5.3)	41.3 (25.5)	0.309
Time since diagnosed with COVID-19 in months, mean (SD)	10.1 (3.4)	10.6 (2.9)	0.489
Symptoms during acute COVID-19, n (%)			
Respiratory symptoms	120 (67.8%)	27 (84.4%)	0.059
Neurological symptoms	81 (45.8%)	22 (68.8%)	0.017
Gastrointestinal symptoms	45 (25.4%)	12 (37.5%)	0.159
Perceived dizziness in long COVID, n (%)			<0.001
Yes	37 (20.9%)	18 (56.3%)	
No	140 (79.1%)	14 (43.8%)	
DHI total score, mean (SD)	7.26 (11.87)	26.9 (27.7)	<0.001
FES-I total score, mean (SD)	20.67 (7.9)	25 (8.8)	<0.001
MFIS total score, mean (SD)	25.4 (21.3)	41.7 (21.4)	<0.001
SF-12 total score, mean (SD)	69.1 (16.3)	52.9 (19.7)	<0.001
Right ear PTA (n = 160), n (%)			0.152
Normal	119 (67.2%)	26 (81.3%)	
Abnormal	10 (5.9%)	5 (15.6%)	
Left ear PTA (n = 160), n (%)			0.043
Normal	122 (68.9%)	26 (81.3%)	
Abnormal	7 (4%)	5 (15.6%)	

BMI: body mass index; DHI: Dizziness Handicap Inventory; PTA: pure-tone audiometry; FES-I: Falls Efficacy Scale International; MFIS: Modified Fatigue Impact Scale; SF-12: Medical Outcomes Study Short Form 12.

**Table 5:** Regression analysis of having hearing loss in long COVID (N = 209).

	Estimates	Standard Error	t Value Wald?	*p* Value
Constant	−1.883	0.629	8.966	0.003
Age	−0.035	0.023	2.341	0.966
DHI total score	0.038	0.010	15.61	<0.001
Neurological symptoms	0.862	0.434	3.946	0.047

DHI: Dizziness Handicap Inventory.

## Data Availability

Data are unavailable due to privacy and ethical restrictions.
